# Cardiomyopathy development protection after myocardial infarction in rats: Successful competition for major dihydropyridines’ common metabolite against captopril

**DOI:** 10.1371/journal.pone.0179633

**Published:** 2017-06-21

**Authors:** Katarzyna A. Mitręga, Adrianna M. Spałek, Jerzy Nożyński, Maurycy Porc, Magdalena Stankiewicz, Tadeusz F. Krzemiński

**Affiliations:** 1 Silesian Centre for Heart Diseases, Zabrze, Poland; 2 Chair and Department of Pharmacology, Medical University of Silesia, Zabrze, Poland; Indiana University School of Medicine, UNITED STATES

## Abstract

During the last 25 years angiotensin-converting enzyme inhibitors spectacularly conquered the field of cardiovascular diseases therapy. Nevertheless, lack of new studies concerning side effects associated with their chronic administration seems to be rather confusing. In our previous research, we proved that the main furnidipines’ metabolite (M-2) possess multiple cardioprotective actions. Currently, we compared effects of post-infarction long-term oral treatment with M-2 and captopril on hemodynamic parameters and “ischemic cardiomyopathy” development in rats. Myocardial infarction was evoked by permanent left anterior descending coronary artery occlusion for 35 days. Surviving rats were treated with captopril (2 × 25 mg/kg) or M-2 (4 mg/kg) from 6^th^– 35^th^ day. At 35^th^ day rats’ hearts were tested on working heart setup, where following parameters were measured: heart rate, preload pressure, aortic systolic and diastolic pressures, aortic maximum rise and fall, aortic and coronary flow, myocardial oxygen consumption and oximetry in perfusate. Subsequently, heart tissue specimens were assessed during morphological estimation. Captopril caused significant heart rate increase and markedly diminished preload pressure in comparison to M-2. Both drugs evoked essential aortic pressure increase. Aortic flow was significantly decreased after M-2, whereas captopril increased this parameter in comparison to M-2. Both agents caused marked coronary flow increase. Morphologic examination in captopril revealed cardiomyopathic process in 70% of hearts, whereas in M-2 this value reached 30%. Neovascularization of post-infarcted myocardium was visible only after M-2 therapy. Concluding, M-2 presented itself as more attractive agent in long-term post-infarction treatment by preventing cardiomyopathy development, angiogenesis stimulation and preserving cardiac performance.

## Introduction

The leading group of drugs currently recommended as a first-line therapy after myocardial infarction are angiotensin-converting enzyme inhibitors (ACEIs) [1]. In the last 25 years they have gained an important position in preventing heart and vascular remodeling as well as preserving cardiac function [2–3]. Moreover, application of therapeutics from this class is strongly associated with patients’ less mortality and improved quality of life [4–6].

The worldwide success of ACEIs is related to their multidimensional activity profile. Besides their clear beneficial influence on endocrine compensatory mechanisms (e.g. limitation of aldosterone release, potentiation of bradykinin effects), they are also proved to counteract the sympathetic stimulation of noradrenaline and demonstrate free radical scavenging properties [7–11]. Additionally, latest breakthrough experimental research on captopril suggested this group of agents can also attenuate changes in myocardial gene expression after MI in rats [12].

Despite their many clinical merits, several considerable trials called their efficacy into question [13–15]. What is more, only few human autopsy research concerning the histopathological effect of long-term treatment with ACEIs on post-infarcted myocardium in terms of cardiomyopathy development have been performed, what still makes this aspect far from being conclusive [16].

Since ACEIs have become a “panacea” in the cardiovascular diseases therapy, -*prils* have been treated as an exhausted topic and nowadays nobody is dealing with the potential side effects associated with their chronic consumption. Accordingly, the aim of following research is to, at least partially, fill this gap as well as attract attention to this neglected issue.

Furnidipine, as well as other dihydropyridines derivatives, is proved to protect the heart from stunning, ischemia and experimental atherosclerosis [17–22]. Furthermore, several studies reported their favourable role in infarct size reduction [23–26]. Due to the ability of L-type calcium channel inhibition and differentiated cardiac depressive action [20–22,27–29], their main therapeutic indications nowadays are hypertension and certain specific forms of angina pectoris [30].

Since it was clarified, M-2 is also a common metabolite present in degradation pathways of many widely used dihydropyridines (including nifedipine), outcomes of our investigations with this agent supply new outlook not only on the effects of M-2 itself, but on this whole group of drugs as well [31–34]. Our former research with M-2 conducted on various experimental *in vivo* and *ex vivo* rat models established its beneficial effects on mortality [31,34], ischemia- and reperfusion-induced lethal arrhythmias [31,33–34] as well as hemodynamic parameters (e.g. blood pressure or coronary flow) [33–34].

Proceeding these investigations, we performed another experiment which aim was to find whether the M-2 could protect, or delay, post-MI cardiomyopathy in rats and establish the most optimal treatment period [35]. Morphologic examination of specimens collected from infarcted rats’ hearts treated with M-2 in dose of 4 mg/kg daily revealed that long-term oral therapy (between 6th– 35^th^ day post-MI) surprisingly guaranteed full protection from “ischemic cardiomyopathy” development. Furthermore, the revitalisation of the vessels and infarcts scars as well as intensification of angiogenic events and inhibition of cardiomyopathic remodeling were clearly visible.

Considering the all promising results with M-2, we consequently decided to confront it with the still widely used and at the same time being the reference drug in clinical trials—captopril (2 × 25 mg/kg) [2,36] in the same regime model i.e. combined model of experimental MI with subsequent test on the standardized working heart (WH) setup followed by decisive histopathological examination.

## Materials and methods

### Experimental animals

The experiments were conducted with male *Sprague-Dawley* rats (*n* = 43) weighing at the beginning approx. 361 ± 55 g (Central Animal Farm, Medical University of Silesia, Katowice, Poland). The animals were housed in individual cages and maintained under standard conditions (ambient temperature 21–23°C, with 12 h dark/light cycle) with *ad libitum* access to food (standard LSM diet, Motycz, Poland) and water. The animals were fasted overnight before the experiment.

The entire study was performed with the approval of the Local Bioethics Committee for Animal Use, Silesian Medical Academy. All experiments were carried out in accordance with NIH regulations of animals care described in “Guide for the Care and Use of Laboratory Animals” (NIH publication, p. 2–107, revised 1996).

### Drugs and reagents used

Following drugs were used in the study: captopril {SQ-14225: (2S)-1-[(2S)-2-methyl-3-sulfanylpropanoyl]pyrrolidine-2-carboxylic acid, MW 217.28; Sigma, Deisenhofen, Germany} and main furnidipines’ metabolite M-2 {[2,6-dimethyl-5-methoxy-carbonyl-4-(2’-nitrophenyl)-pyridine-3-carboxyliquide acid, MW 330.29]; Cermol S.A., Geneva, Switzerland}. For oral administration, captopril and M-2 solutions were prepared respectively in water or 0.4% aqueous dimethylsulfoxide (DMSO) and given in a volume of 5 mL/kg. Unless otherwise stated, all other reagents were of the highest purity and were supplied by Sigma Chemical Co. (Deisenhofen, Germany).

### Experimental models

The entire study was consisted of two successive experimental rats’ models: 1) *in vivo* model of myocardial infarction, according to the method described by Selye et al. [37] and Guendjev [38] with own improvements [39–42] and 2) *in vitro* model of physiological perfusion of the isolated rat heart (working heart, WH) previously described elsewhere [42–43] in accordance to the method described by Neely et al. [44].

#### Experimental infarction in rats

The myocardial infarction was induced by permanent left anterior descending coronary artery (LAD) occlusion for 35 days, this being the same survival time period of these animals. The rats were anesthetized with pentobarbital (60 mg/kg intraperitoneally; *ip*, Sigma, Germany), heparinized (500 IU/100 g body weight *ip*). In order to compare the depth of anesthesia, reflex response to noise and pain induced by the pinching of the limbs and distal portion of the tail, were tested in each rat at the beginning and the end of the experiment as prescribed [45–46]. Rectal temperature was maintained at approximately 38°C.

In brief, the trachea was incised longitudinally and cannulated to allow artificial ventilation. The chest was opened under ventilation with room air (55–60% humidity, 23°C, stroke volume 0.8 ml/100 g of body weight; rate 54 strokes/min with the positive end-respiratory pressure of 1 cm H_2_O; Rodent VENTILATOR-UB 7025, Hugo Sachs Elektronik /HSE/, March, Germany) [47] by left thoracotomy at the fifth intercostal space, the fourth and fifth ribs were sectioned approximately 2 mm from the left margin of the sternum. After opening the pericardium the heart was not exteriorized and a sling (6/0 Prolene 0.7 suture attached to 3/8 circled BV-1 a 9.3 mm atraumatic, reverse cutting needle, EH 7406H, Ethicon GmbH, Norderstedt, Germany) was placed around LAD close to its origin (2 mm below). Then the ligature was passed through the plastic pad (polyethylene, 2 mm OD /0.5 ID/, thickness 0.2 mm). The coronary artery was occluded by applying tension to the ligature while pressing the pad onto the heart surface. Tension was maintained by clamping a climb clip (Titan climb clip, LT-100, Ethicon GmbH, Norderstedt, Germany). Successful occlusion was immediately confirmed by the ischemia-induced alteration in ECG (ST-elevation e.g.) and observation of an arising pale ischemic zone below the climb clip. The ECG was recorded from standard limb leads using needle electrodes and recorded synchronously with the blood pressure curve on a high-speed chart recorder (Line Recorder TZ 4620, Laboratorni Pristroje, Praha, Czech) and displayed in parallel on a digital cardio monitor (CMK 405, TEMED, Zabrze, Poland). At the end of the operating procedure (approx. 15 min) tissues were sutured in layers (4–0 Deklene TM-II, 1.5, D-5427, Ethicon GmbH, Norderstedt, Germany) excluding the pericardium (avoiding heart tamponade). The rats awoke in few hours after closing the thorax. The postoperative mortality rate of all rats was 7% (mainly caused by lethal arrhythmias and circulatory and/or respiratory insufficiency during the first day post-MI). Furthermore, the surviving rats were housed for 35 days.

#### Standardized working heart setup

At 35^th^ day of experiment, the animals were weighted again, heparinized (500 IU/100g body weight, *ip* heparin sodium Polfa, Poland) and anesthetized with pentobarbital (60 mg/kg *ip*). The chest was opened by a left thoracotomy and hearts were rapidly excised together with lungs and arrested by chilling in the beaker with ice-cold modified Krebs-Henseleit bicarbonate buffer (K-H) and weighted. The ascending aorta was cannulated with a steel cannula and perfused according to non—recirculating Langendorff method [48] with a constant perfusion pressure (60 mmHg; equal to afterload used in the WH setup) with a modified K-H buffer [pH 7.4–7.45 at 37°C consisting of the following (in millimolar): NaCl 118, KCl 4.7, NaHCO_3_ 24.88, CaCl_2_ 2.52, KH_2_PO_4_ 1.18, MgSO_4_ 1.64, glucose 11.1, pyruvate 2.0, saturated with 95% O_2_ and 5% CO_2_].

The air temperature around the heart surface was maintained at 37°C by a beaker with a jacket containing water. The lung lobes were subsequently cut out and weighted to obtain wet weight of the heart. In order to convert the Langendorff preparation into a WH mode [44], the veins were ligated close to the surface of the right atrium and the left atrium was cannulated with steel cannula. A plastic cannula was placed in the pulmonary artery to drain the coronary effluent perfusate for *p*O_2_, *p*CO_2_ and pH measurement. Perfusion through the aorta was switched to perfusion through the left atrium. The initial atrial filling pressure was adjusted to 12 mm Hg (preload). The left ventricle ejected the perfusion fluid through an aortic cannula into an overflow system in which the aortic pressure was held at 60 mm Hg (afterload). At the end of the preparation, suction electrodes were attached onto the heart surface for electrogram (EG) recording [49] and *p*O_2_, *p*CO_2_, pH in affluent perfusate were measured (*p*O_2_ > 530 mm Hg just before left atrium). The hearts were allowed to beat spontaneously. All received signals were transmitted through 16-channel A/D converter and stored away every 30s by an IBM compatible computer with the own necessary software for data acquisition and elaboration (off-line).

**Fig 1:**
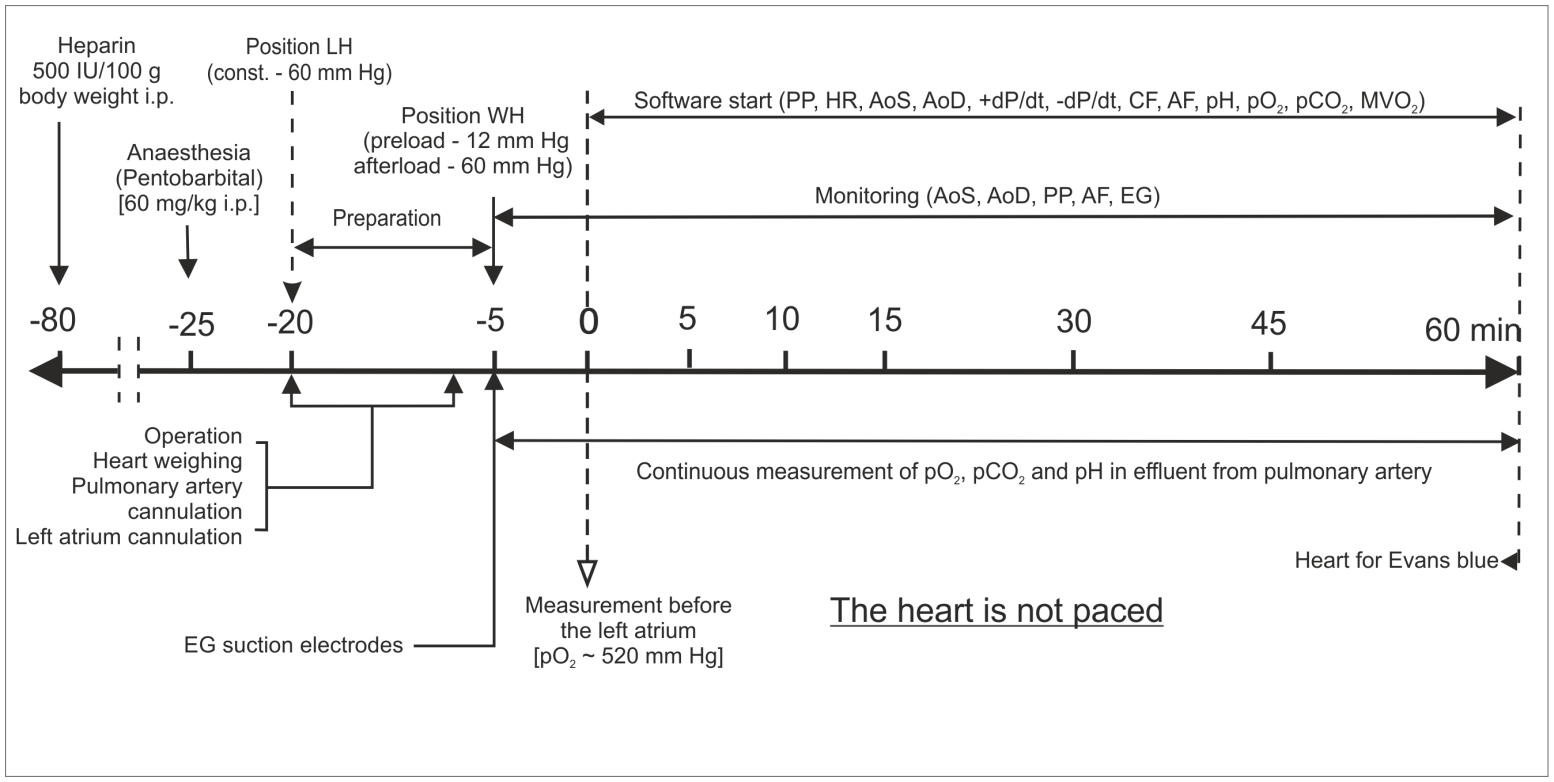
Schedule of the protocol study in the model of experimental myocardial infarction in rats. LH indicates Langedorff mode and WH the working heart mode. Aortic systolic pressure (*AoS*), aortic diastolic pressure (*AoD*), aortic flow (*AF*), coronary flow (*CF*), maximum rate of aortic systolic pressure increase (+*dP/dt*), maximum rate of aortic systolic pressure decrease (-*dP/dt*), oxygen partial pressure (*pO*_*2*_) and carbon dioxide partial pressure (*pCO*_*2*_) and pH values in pulmonary effluent, myocardial oxygen consumption (*MVO*_*2*_), preload pressure (*PP*), heart rate (*HR*).

The following parameters were measured during 60 min of the WH experiment every 30s: heart rate (*HR*, calculated from EG curve), left atrial filling pressure (*PP*, preload pressure), aortic systolic (*AoS*) and diastolic (*AoD*) pressures, aortic maximum rise (+*dP/dt*) and fall (-*dP/dt*) of the first pressure derivative calculated respectively by pressure transducers (ISOTEC, HSE, March-Hugstetten, Germany) connected to the PLUGSYS module (HSE), aortic flow (*AF*, measured by a flow detector connected to electromagnetic flow meter, Electromagnetic Flowmeter, Narco Bio-systems, Houston, TX, USA), coronary flow (*CF*, calculated as the difference between total flow amount of perfusate pumped into the left atrium per time unit and *AF*), *p*O_2_, *p*CO_2_ and pH in pulmonary effluent (Plastomed 450, HTL, Warszawa, Poland) and myocardial oxygen consumption (*MVO*_*2*_, calculated according to Zander and Euler [50] using the formula: *CF*/g wwt × (affluent *p*O_2_ –effluent *p*O_2_) × c × 100 g wwt—heart wet weight c = 0.0240 (Bunsen oxygen solubility for K-H solution at 37°C). Schedule of the experimental protocol was shown in Fig 1.

### Biochemical estimation in blood serum

At 35^th^ day of experiment, 1 mL of rats’ blood was collected directly from aortic arch and without heparinizing dissolved in saline (1/1 vol./vol.) for biochemical estimation prior to WH study. Blood samples were analyzed spectrophotometrically (SPECOL 220, VEB Carl Zeiss Jena, Germany) for the concentration of: α-amylase (U/L, 578 nm), bilirubin (mg/dL, 340 nm), creatine kinase (*CK*, U/L, 340 nm), creatinine (mg/dL, 340 nm), glucose (mg/dL, 340 nm), glutamic oxaloacetic transaminase (*GOT*, U/L, 340 nm), glutamic pyruvic transferase (*GPT*, U/L, 340 nm) and urea (mg/dL, 530 nm) [51] (see Table 1).

**Table 1:** The influence of long-term oral treatment (6^th^– 35^th^ day) after experimental myocardial infarction with captopril (2 × 25 mg/kg) or M-2 (4 mg/kg) on biochemical parameters measured in rats’ blood serum.

	Number of animals	Amylase [U/l]	CK [U/l]	Creatinin [mg/dl]	Glucose [mg/dl]	GOT [U/l]	GPT [U/l]	Urea [mg/dl]
**Intact**	*n* = 9	4 323.30 ± 241.90	216.40 ± 87.70	<dl	139.60 ± 6.92	<dl	21.35 ± 1.29	53.46 ± 2.68
**DMSO**	*n* = 5	2 917.50 ± 553.77	46.13 ± 15.62	<dl	130.33 ± 14.87	<dl	19.35 ± 2.37	46.28 ± 6.78
**Captopril**	*n* = 12	3 696.25 ± 615.04	152.62 ± 253.62	0.18 ± 0.26	116.65 ± 14.90	44.18 ± 3.38	26.63 ± 1.78	64.2 ± 3.65^○^
**M-2**	*n* = 9	2 469.00 ± 433.83^##^	59.15 ± 51.84	0.06 ± 0.18	116.33 ± 15.43	36.47 ± 11.77	10.62 ± 4.85^##^	49.34 ± 6.72^#^

The results are presented as mean ± standard deviation. Non-parametric Kruskal-Wallis ANOVA test was used for all comparisons. Values marked with ○*P* < 0.01 are significantly different from DMSO group. Values marked with ^*#*^*P* < 0.01 and ^*##*^*P* < 0.001 are significantly different from captopril group. <dl—below detection limit; CK—creatine kinase; GOT—glutamic oxoloacetic transaminase; GPT—glutamic pyruvic transferase

### Experimental groups

The rats which survived with myocardial infarction were randomly divided into four groups: captopril (*n* = 12), M-2 (*n* = 9), DMSO (*n* = 5) and control group (*n* = 8). Drugs were administered orally in doses: 2 × 25 mg/kg (captopril), 4 mg/kg (M-2), 0.4% DMSO and 0.9% NaCl (control group) in the volume of 5 ml/kg from day 6^th^ until the end of the experiment on 35^th^ day. The choice of administration period as well as oral doses of M-2 and captopril was based on hemodynamical and morphological findings reported in various research [2,28,32,35–36]. For the biochemical estimation, in order to obtain the physiological values of parameters mentioned above, the intact group (*n* = 9) was added to the trial.

### Determination of myocardial infarct size

Before morphological examination, each removed heart was perfused 5 min through the cannula inserted into aorta with 1 mL of Evans blue (2%; perfusion pressure 135 cm H_2_O). Then it was frozen at -20°C for 5–20 min, cut into 1.5–2.0 mm sagittal sections and immersed in 1% solution of 2,3,5-triphenyltetrazolium chloride (TTC, Sigma, Poole, U.K.) in phosphate buffer (20 mM, pH 7.4) at 37°C for 5–15 min. The white area without Evans blue and TTC was considered as infarcted necrotic myocardium, the blue area-normal myocardium and the red area (stained by TTC)-ischemic myocardium. The myocardium was dissected according to its colors and weighed separately. The percentage ratio of the weight of infarcted necrotic myocardium to that of total ischemic myocardium (infarcted necrotic myocardium and ischemic non-necrotic myocardium) was calculated and designated as the infarct size [52]. The area of infarct in all survived animals (*n* = 43) was 54 ± 6.8%. All treatments and measurements were performed by an experimenter blind to the treatment group.

### Morphologic examination

The infarct-related areas of the heart tissue were fixed in 4% of formaldehyde adjusted to pH 7.4. After routine processing through graded alcohols and xylene, the tissue was embedded in paraffin. Thin 5 μm paraffin sections of infarct-related areas of each sectioned heart were stained with hematoxylin-eozin and Masson’s trichrome stains for light microscopic analysis. Histological slices (5 μm thick) were scanned with EDHISTECH slide scanner (3DHISTECH Kft. Budapest, Hungary) with 20× objective and stored. The images were comparatively evaluated under low and high magnification. In order to exclude the impact of operating procedures on results, intact (sham-operated) group (*n* = 9) was also examined.

Cardiomyopathic features were determined according to the standards established by Olsen et al. [53–54] as: dilation of cardiomyocytes, cardiomyocytes stretching, hypertrophic and/or cardiomyocyte bizarre nuclei, dispersed fibrosis, non-active inflammatory infiltrations.

The same procedure was used for morphologic analysis of the specimens from other organs (liver, kidneys and spleen) in all groups.

Intensification of inflammatory processes in studied material was assessed with our own classification (established as a working hypothesis) and defined as the following grades: single inflammatory cells (0), inflammatory cells in clusters up to 5 cells in nearly 50% of specimen (1), inflammatory cells in clusters over 5 cells or multiple inflammatory aggregates in almost all studied specimen (2). Please see Table 2.

**Table 2:** Inflammatory infiltrations in studied material.

	Number of animals	Minimum	Maximum	Mean range
**Intact**	*n* = 9	0	0	0
**Control**	*n* = 8	0	2	0.6
**DMSO**	*n* = 5	0	1	0.3
**Captopril**	*n* = 12	0	2	1.2
**M-2**	*n* = 9	0	2	0.8

Non-parametric Kruskal-Wallis ANOVA test was used for all comparisons.

Myocardial fibrotic score, based on the modification of the Bilingham classification [55] was determined as: thin collagen fibers between cardiomyocytes (0), collagen fibers separate cardiomyocytes below doubled cardiomyocyte diameter, and/or small areas of fibrosis are clearly visible (1), collagen fibers separate cardiomyocytes over doubled cardiomyocyte diameter, and/or numerous areas of fibrosis are clearly visible (2). Please see Table 3.

**Table 3:** Myocardial fibrosis in studied material.

	Number of animals	Minimum	Maximum	Mean range
**Intact**	*n* = 9	0	0	0
**Control**	*n* = 8	0	2	1.7
**DMSO**	*n* = 5	0	1	0.8
**Captopril**	*n* = 12	0	2	1.1
**M-2**	*n* = 9	0	2	0.5*

Non-parametric Kruskal-Wallis ANOVA test was used for all comparisons. Values marked with *P < 0.05 are significantly different from control group.

### Statistical analysis

The hemodynamic results measured every 30 s during WH study were averaged in order to receive mean values for each minute. Results of biochemical study are presented as mean ± standard deviation.

The Kruskal-Wallis ANOVA test was used to estimate the significance of measured parameters among all groups in WH study, biochemical analysis and morphologic examination (inflammatory infiltrations, myocardial fibrosis) as well as to assess the differences in wet hearts’ and rats’ weight.

The chi-square-test (χ^2^; Yates) was used to estimate the significance between the incidence of cardiomyopathy development in all comparisons.

In all cases, differences were considered significant at *P* < 0.05.

## Results

### Hemodynamics on working heart study

In the preliminary study (performed exactly in the same protocol) no side effects in the hemodynamic parameters in the sham groups treated with captopril, M-2 and DMSO were found.

**Fig 2:**
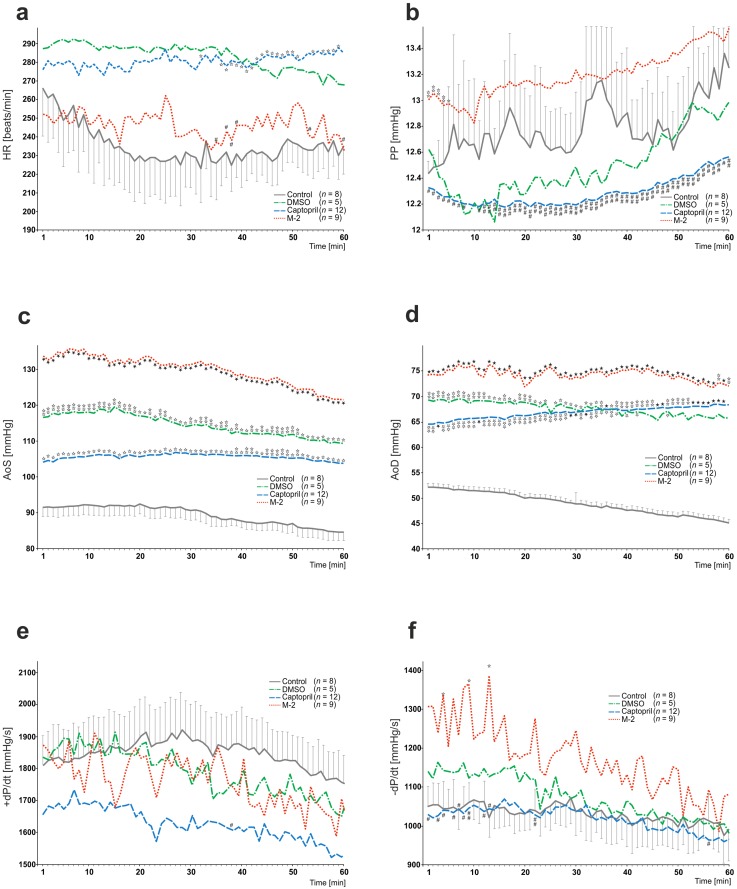
The influence of long-term oral treatment (6^th^– 35^th^ day) with captopril (2 × 25 mg/kg) and M-2 (4 mg/kg) after experimental myocardial infarction (MI) on: (A) heart rate (HR), (B) preload pressure (PP), (C) aortic systolic pressure (AoS), (D) aortic diastolic pressure (AoD), (E) aortic maximum rise of the first pressure derivative (+d*P*/d*t*) and (F) aortic maximal fall of the first pressure derivative (-d*P*/d*t*), tested in working heart setup. Data are expressed as mean values for each minute. For simplicity sake, standard error of the mean is presented only in control group. Non-parametric Kruskal-Wallis ANOVA test was used for all comparisons. Values marked with ✩*P*<0.05, ✩✩*P*<0.01 or ★*P*<0.001 are significantly different from control group. Values marked with ○*P*<0.05 or ○○*P*<0.01 are significantly different from DMSO group. Values marked with ^*#*^*P*<0.05 or ^*##*^*P*<0.01 are significantly different between captopril and M-2 group.

**Fig 3:**
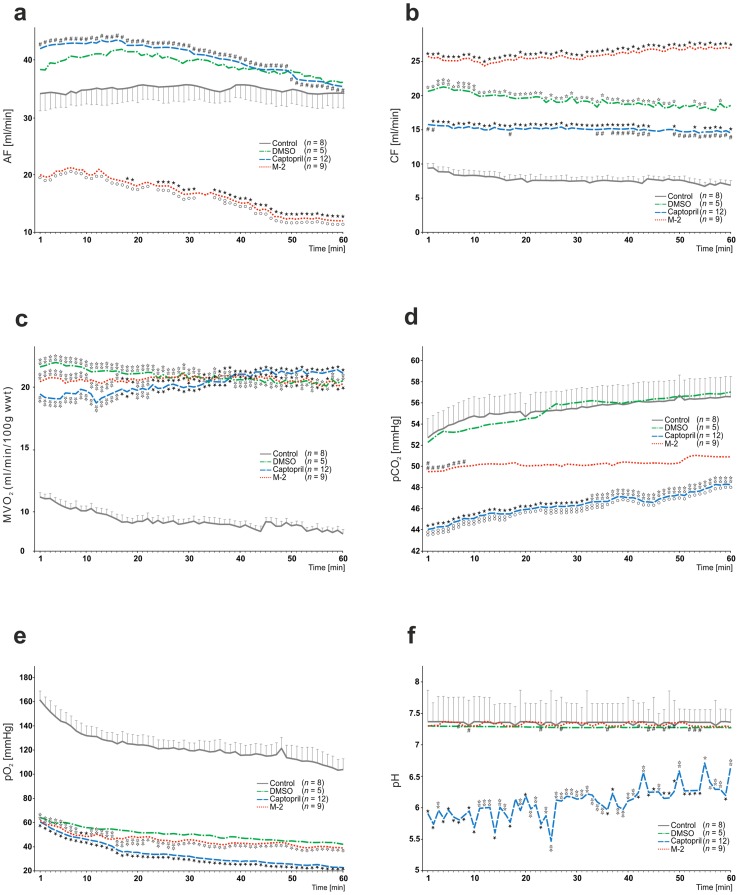
The influence of long-term oral treatment (6^th^– 35^th^ day) with captopril (2 × 25 mg/kg) and M-2 (4 mg/kg) after experimental myocardial infarction (MI) on: (A) aortic flow (AF), (B) coronary flow (CF), (C) myocardial oxygen consumption (MVO_2_), (D) partial pressure of CO_2_ in perfusate (*p*CO_2_), (E) partial pressure of O_2_ in perfusate (*p*O_2_) and (F) pH, tested in working heart setup. Data are expressed as mean values for each minute. For simplicity sake, standard error of the mean is presented only in control group. Non-parametric Kruskal-Wallis ANOVA test was used for all comparisons. Values marked with ✩*P*<0.05, ✩✩*P*<0.01 or ★*P*<0.001 are significantly different from control group. Values marked with ○*P*<0.05 or ○○*P*<0.01 are significantly different from DMSO group. Values marked with ^*#*^*P*<0.05 or ^*##*^*P*<0.01 are significantly different between captopril and M-2 group.

During 60 min of this experiment many relationships between captopril or M-2 *vs*. control/DMSO groups as well as between drugs themselves were found (Figs 2 and 3).

Among all studied groups, captopril caused the most significant *HR* increase in comparison to control and in part to M-2, however this effect was observed only in the second half of the experiment (*P* < 0.05). When compared to M-2, ACEI effectively diminished the *PP* values during the whole study (*P* < 0.01). Although the M-2 > captopril > DMSO evoked marked the *AoS* and *AoD* increase this action was the most pronounced after M-2 treatment (*P* < 0.001 *vs*. *P* < 0.01 *vs*. *P* < 0.05, respectively). Neither of drugs considerably affect on +*dP/dt* values, nevertheless captopril slightly decreased -*dP/dt* in comparison to M-2 (*P* < 0.05). Importantly, the *AF* was significantly decreased after M-2 treatment (*P* < 0.001) in comparison to control and DMSO groups, whereas captopril caused marked *AF* increase in relation to main furnidipine metabolite during entire experiment (*P* < 0.01). As expected, all agents evoked the essential *CF* increase, when compared to control. It should be emphasized that this effect was the most pronounced in absolute values after M-2 treatment. However captopril also caused this *CF* increase, it was less marked in comparison to M-2 (*P* < 0.05 between captopril and M-2 groups). In consequence, captopril and M-2 caused significant the *MVO*_*2*_ increase (*P* < 0.01).

It should be added that this effect strongly correlates with different wet heart weight/body weight (HW/BW) ratio among studied groups: intact HW/BW: 3.99 × 10^−3^; control HW/BW: 4.03 × 10^−3^; DMSO HW/BW: 4.11 × 10^−3^; M-2 HW/BW: 4.83 × 10^−3^; captopril HW/BW: 3.23 × 10^−3^. Statistical analysis of this data revealed significance between captopril *vs*. intact group (*P* < 0.05) as well as captopril *vs*. M-2 groups (*P <* 0.0001).

Unlikely to others, captopril most effectively diminished *p*CO_2_ values in perfusate. During whole study captopril and M-2 caused significant the *p*O_2_ decrease (*P* < 0.05). Finally, among all studied groups captopril markedly diminished the pH values in comparison to M-2 (*P* < 0.05) and control groups (*P* < 0.01).

### Biochemical estimation in blood samples

Long-term oral treatment (6th– 35^th^ day after MI) with captopril and M-2 did not markedly influence on most measured biochemical parameters including CK. Surprisingly, captopril significantly increased urea concentration in comparison to DMSO group (*P* < 0.01). What is more, M-2 in relation to captopril strongly reduced concentration of α-amylase, GTP (*P* < 0.001 respectively) and, to lesser extent, urea (*P* < 0.01) in rats’ blood serum (Table 1).

### Histopathological study

In the preliminary study (performed exactly in the same protocol) no side effects in the histopathological examination in the sham groups treated with captopril, M-2 and DMSO were found.

**Fig 4:**
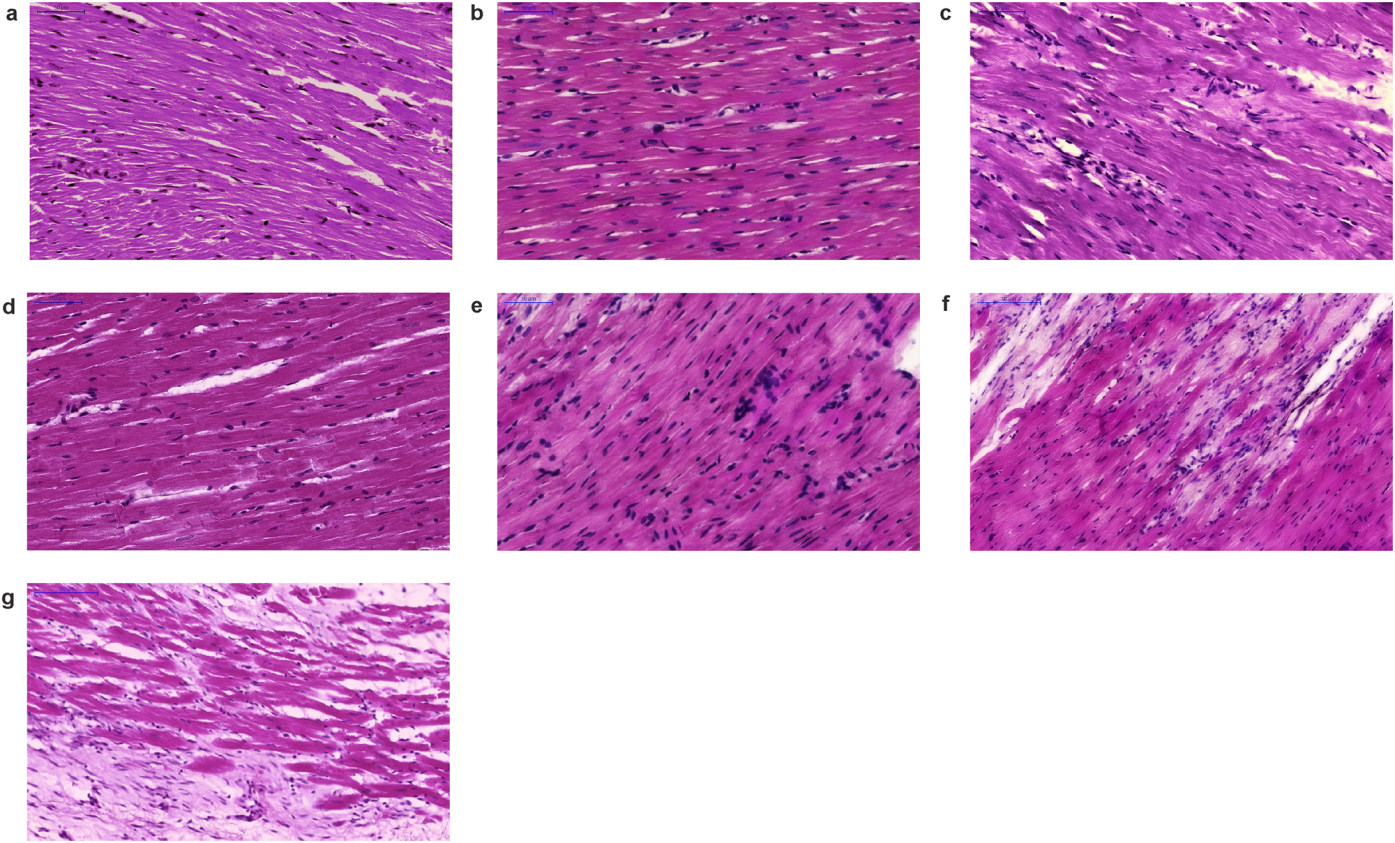
Representative histological findings of myocardial tissue evaluation at 35^th^ day post-infarction after long-term oral treatment (6^th^– 35^th^ day) with captopril (2 × 25 mg/kg) or M-2 (4 mg/kg) (hematoxylin and eozine-stained). (A) Intact specimen of sham-operated heart, (B-C) Control 0.9% NaCl, (D) DMSO group, (E-F) Captopril group, (G) M-2 group (scanned with 20× objective, bar represents 50–100 μm).
(A)Closely arranged cardiomyocytes with comparable nuclei. Normal myocardial tissue (magnification 30×).(B)Non-uniform cardiomyocytes with enlarged, rounded nuclei suggesting early cardiomyopathic process. Interstitial space contains some lymphocytes (magnification 30×).(C)Evident cardiomyopathic pattern with perivascular inflammatory infiltrations composed of lymphocytes and larger macrophages (magnification 30×).(D)Myocardial tissue morphology resembles intact group specimens (magnification 30×).(E)Typical cardiomyopathic pattern with numerous, dense inflammatory infiltrations with evident histiocytes, lymphocytes and other cells (magnification 30×).(F)Advanced cardiomyopathy with present fibrotic focai (magnification 20×).(G)Moderate cardiomyopathic pattern with lesser inflammatory infiltrations in comparison to captopril group. Regular fibrotic process (magnification 20×).

The most striking observations of our entire study were seen in morphologic examination (Fig 4).

No histological abnormalities in the hearts of intact animals were found (Fig 4A).

The area of infarct in all survived animals (*n* = 43) was 54 ± 6.8%. There was no significant differences in the infarcted necrotic myocardium area among studied groups.

At 35^th^ day post-experimental MI, typical cardiomyopathic morphology was visible in all of the control group hearts. Fibrotic scars with some inflammatory infiltrates were predominant in the myocardial tissue. Presented vessels were remnant with associated perivascular accumulation of various cells including lymphocytes and macrophages (Fig 4B and 4C). Loss of cross striation was seen in some areas as well.

Myocardial tissue in DMSO group mainly resembled pictures observed in intact rats’ hearts (Fig 4D).

**Fig 5:**
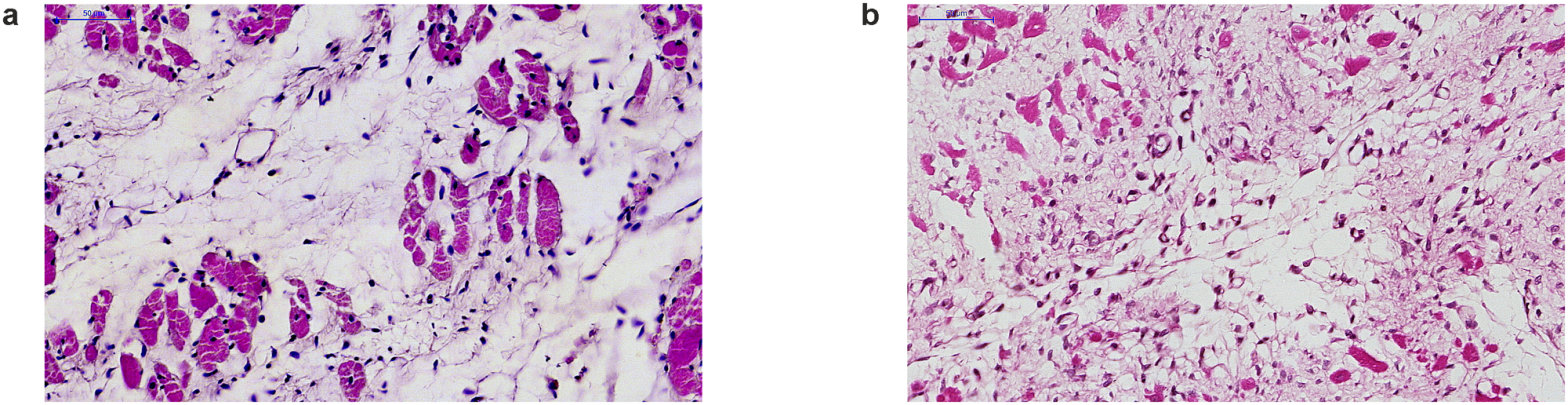
Representative histological findings of epicardial vessels evaluation at 35^th^ day post-infarction after long-term oral treatment (6^th^– 35^th^ day) with captopril (2 × 25 mg/kg) or M-2 (4 mg/kg) (hematoxylin and eozine-stained). (A) Captopril group, (B) M-2 group (scanned with 20× objective, magnification 30×, bar represents 50 μm).
(A)Small vessels composed of flattened endothelial cells observed inside fibrotic area.(B)Multiple, small capillary vessels composed of enlarged endothelial cells. Predominant part of endothelial cells reveals basophilic cytoplasm suggesting progressive neovascularization process.

Most of captopril group specimens revealed advanced cardiomyopathic process. Myocardial tissue was hypertrophied and presented evident signs of fibrosis with different amount of lymphocytic infiltrations (Fig 4E and 4F). Observed infarct scars were scanty vascularized by few differentiated vessels (Fig 5A).

Surprisingly, cardiomyopathic morphology in M-2 treated group was absent in most of the cases. Only few hearts in this group revealed cardiomyopathic features with slight fibrosis in myocardial tissue. Furthermore, unlike to other groups, the domination of young capillary vessels near infarct scars (Fig 5B) as well as the definite reduction of inflammatory infiltrate (Fig 4G) were apparent and astonishing. What is more, it should be emphasized that evident signs of angiogenesis were present (descended capillaries with intensified cells proliferation). Accordingly, it could be stated that neovascularization of post-infarcted myocardium was visible only after M-2 treatment.

The overall statistic score of inflammatory infiltrations presented as Table 2 did not reveal any significant differences among studied groups. However, the intensification of myocardial fibrosis (Table 3) in M-2 group was markedly diminished in comparison to the control group.

The morphologic examination of the specimens collected from other organs (liver, kidneys and spleen) revealed intensified hyperemia in captopril and M-2 groups, but they did not differ from controls in general (data not shown).

The statistical estimation of heart slices from all groups confirmed the observations stated above. Summing up, almost 70% of specimens from captopril group revealed cardiomyopathy development, whereas in M-2 group this value reached just 30% (Table 4). Moreover, statistical analysis indicated that M-2 treatment significantly diminished the number of cardiomyopathy presence in comparison to control group, whereas after captopril therapy this phenomenon was not exhibited.

**Table 4:** Incidence of cardiomyopathy development in rats treated orally with captopril (2 × 25 mg/kg) or M-2 (4 mg/kg) from 6^th^ to 35^th^ day after experimental myocardial infarction.

	Number of animals	Hearts with cardiomyopathy	Hearts with cardiomyopathy [%]
**Intact**	*n* = 9	0	0
**Control**	*n* = 8	8	100
**DMSO**	*n* = 5	2	40
**Captopril**	*n* = 12	8	66.67
**M-2**	*n* = 9	3*	33.33

The chi-square-test (χ^2^; Yates) was used to estimate the significance between the incidence of cardiomyopathy development in all comparisons. Values marked with *P < 0.05 are significantly different from control group.

## Discussion

Following the results presented above, captopril and M-2 showed themselves as multi-active agents with powerful, diverse influence on post-infarcted rats’ myocardium. After long-term oral treatment (6th– 35^th^ day after experimental MI) both drugs evoked considerable changes at two different levels of examination: WH, illustrating hemodynamical hearts’ function after MI, and *post-mortem* histopathological study.

### M-2

Basing on the observations from WH study (Figs 2 and 3), it could be concluded that M-2 mainly caused the flow elevation through small coronary vessels rather than intensification of aortic flow. It seems obvious that *CF* increase gives opportunity for amelioration of ischemic areas and may be beneficial in heart healing after infarction as well. Furthermore, significant fall in *AF* as well as no marked impact on *HR* indicate on sparing influence of M-2 on damaged myocardium.

The molecular mechanism of M-2 action on hemodynamic parameters is probably composed of its’ many various properties such as: L-type calcium channel blocking, outward potassium ATP-dependent channels activation and nitric oxide (NO) donation [28,32–33].

The lack of cardiodepressive action of M-2 is attributed to its stronger affinity to outward potassium ATP-dependent channels than calcium channels [28,33,56]. Previous experiments have shown the reduction in anoxia-induced shortening of action potential (> 90% reduction) [33] indicate on M-2 direct role as an outward potassium ATP-dependent channels gating protector. Additionally, revealed decrease of the intracellular free calcium ion concentration during hypoxia in non-stimulated isolated guinea pig cardiomyocytes after M-2 administration could be explained due to an effect on an alternative calcium entry via modified sodium channels or via sodium/calcium ions exchange [28]. Besides its known activities, it might be speculated that M-2 participates in cardiac remodeling through NO release. We have previously reported that the local expression of inducible nitic oxide synthase (iNOS) as well as tumor necrosis factor (TNFα) and vascular endothelial growth factor (VEGF) in inflamed border zone of infarcted myocardium is related to the depression of cardiomyocytes contractility leading to progressive development of heart failure [40–41]. In addition, the excess of NO (either produced by iNOS or released from M-2), continuously present till day 35^th^ after MI [40–41], can be cytotoxic to cardiomyocytes and endothelium [57], however NO is also supposed to stimulate amelioration of the coronary bed after infarction.

In our previous study, we established that administration period of M-2 between 6^th^-35^th^ day post-MI in the dose of 4 mg/kg daily completely protected ischemic heart tissue from cardiomyopathy development in rats [35]. Moreover, our highlights presented in the overview of morphological changes in non-treated rats’ infarcted myocardium revealed that these processes are quite analogous in man, however some of them may proceed faster [32]. Accordingly, the necrotic cardiomyocytes as well as inflammatory infiltrations started to diminish and be replace by young connective tissue at day 6^th^, whereas fully evident cardiomyopathic morphology was apparent and complete at 35^th^ day after experimental MI. This period can be considered as appropriate time-frame for effective therapeutic protection against the post-MI “ischemic cardiomyopathy” development in rats.

In present research, the favorable hemodynamic results after long-term therapy with M-2 proved to be a reflection of enhancing heart condition observed in rats’ *post-mortem* examination (Figs 4 and 5). Accordingly, the histopathological analysis with significances (Table 4), confirmed the cardioprotective role of M-2 long-term treatment through breaking cardiomyopathic rebuilding after MI. This major dihydropyridines’ common metabolite positively affected on hearts post-infarction recovery mainly through the angiogenesis process stimulation, which is supposed to be a leading vector in revitalization of infarct scar. As stated above, the observed small amount of inflammatory infiltrations, considered as a source of TNFα, interleukines and etc., can be meaningful aspect in preserving cardiac efficiency.

### Captopril

Results obtained in working heart study (Figs 2 and 3) revealed unfamiliar chronotropic activity of captopril. This marked increase in *HR* (in second half of the experiment) is not typical for ACEIs and certainly overloads damaged heart muscle. We suppose, this autonomic effect might be explained as a compensatory reaction to the relatively low values in the aortic systolic and diastolic pressures. In turn, significant preload pressure decrease that follows captopril long-term therapy is assumed to be a beneficial hemodynamic effect. In clinic, it can be explain by an effective fall in the systemic vascular resistance due to the lack of vasoconstrictor angiotensin II as well as ACEIs ability to attenuate sympathetic activity of noradrenaline [9,58]. Consequently, the fall in *PP* values plays the essential role in cardiac output elevation associated with better cardiac performance. Interestingly, our observations indicate on captoprils’ differentiated influence on various vascular beds. Similarly to M-2, its action is associated with coronary flow increase, however captopril did not affect the *AF* values in comparison to control. It has been proposed, this contradictory reactions can be due to different angiotensin II receptors existing in systemic and coronary vessels [59]. Accordingly, small coronary vessels seem to be the ones more sensitive to the inhibition of renin-angiotensin system. This proposed working hypothesis can potentially elucidate the surprising hypertensive effect of captopril long-term administration in our experiment. Moreover, captopril is also proved to stimulate coronary vasodilatation through enhanced prostaglandins synthesis as well as the inhibitory effect of prostaglandins on noradrenaline release [60]. Lastly, spontaneous acidification of post-infarcted myocardium after captopril long-term therapy can be potentially one of the ways it influence on endocrine system. A body of evidence suggests that even slight fall in pH, independently from vessels dilatation, is responsible for the activation of kinin system [61], which is a well-known ACEIs property [62].

In summary, beneficial hemodynamic performance did not correlate with heart tissue changes observed after oral long term captopril treatment, while histopathological examination revealed the most intensified cardiomyopathy remodeling among studied groups (Table 4).

Since ACEIs became a revolution in cardiovascular diseases therapy, their detrimental influence on preventing heart muscle remodeling has been strongly suggested in the last 25 years [2–3]. Their advantageous effect on infarct size reduction [63] and post-MI cardiac weight regression [64] has been observed even in the low doses of ACEIs, which even did not reduce blood pressure values [65]. All these salutary effects of ACEIs on remodeling inhibition are dedicated to their unique but still enigmatic activity profile. The most considerable theory indicates on the locally synthesized angiotensin II in the failing myocardium as the major mitogenic promotor of the left ventricular dilatation and vascular smooth muscle cells hypertrophy [11,65]. However, considering the hypothesis that angiotensinogen expression is normalized after 25 days post-MI in rats [66], the indirect stimulus for the induction of cardiac remodeling seems yet uncertain. Some authors even speculated the ACEIs target goes much deeper than just influencing renin-angiotensin-aldosterone system and potentiating bradykinin activity. Accordingly, it has been reported that the inhibition of cardiac hypertrophy and improvement in cardiac function after post-MI captopril treatment is dependent on the normalization in gene expression coding e.g. monoamine oxidase, cytochrome P450, cytosolic epoxide hydrolase and some others [12]. Although various hypothesis dealing with ACEIs mechanisms of action have been investigated, including their anti-inflammatory or anti-scavenging properties [8,10,36], some of the mentioned above still remain disputable. Numerous inflammatory infiltrations observed in our histopathological findings contradict the captoprils’ ability to inhibit neutrophils chemotaxis, which are responsible for harmful superoxide anions release [36]. Moreover, even if possible, all of these potentially cardioprotective ACE-independent actions of captopril may not be share by most of the other ACEIs due to the lack of a sulfhydryl group [36].

Simultaneously, the ACEIs clinical status of versatile cardiovascular drugs has been also undermined in the various large studies [13–15]. Some authors reported that their efficacy remains insecure, especially, when it comes to prolonged, global ischemic episodes, myocardial stunning or cardioplegia [10]. These conclusions were based on the results from experiment conducted using spontaneously hypertensive rats, which revealed that although post-MI long-term quinapril administration stabilized hemodynamic parameters, it did not reduce ventricular remodeling and even impaired scar healing in large infarcts at the same time [13]. These observations fully harmonize with our current outcomes. However ACEIs are proved to be drugs, which prolong survival in patients with both asymptomatic and symptomatic congestive heart failure [58], their efficacy has been undermined in patients with stable coronary artery disease [14] or early after coronary artery bypass grafting in whom the rate of adverse cardiovascular events were low [15]. Nevertheless, the authors of these trials did not recommend to withdrawn or limit ACEIs intake from standard therapy in mentioned indications.

### Clinical outlook

During last 25 years ACEIs spectacularly spread over many fields of medicine, giving the impression that dreams about perfect cardiovascular drugs finally came true. Accordingly, it seems that the aspects of potential dangers connected with their long-term administration remain omitted. The hidden aim of current research was to drop a hint on the effects of long-term oral administration of the reference agent of this major class of cardiovascular drugs. Regardless of all their undeniable credits, it is important to maintain pharmacovigilance and constantly be aware that even potentially the best of therapeutics are not free of serious side effects, especially when administrated chronically. Once again, it is worth emphasizing that additional insight in the histopathological structure of experimental post-infarcted myocardium after captopril long-term treatment allowed to observe unfavorable heart tissue changes (i.e. hypertrophy, fibrosis, lack of neovascularization and cardiomyopathy development), which remain hidden during typical therapy at the same time being responsible for consequent deterioration of the heart function.

## Final conclusion

Summing up, the results of our entire study establish beneficial cardioprotective influence of long-term oral treatment with M-2 preventing “ischemic cardiomyopathy” development after infarction (histopathological examination) as well as preserving cardiac performance (working heart study). In details, the major vectors of the favorable effects evoked by main furnidipines’ metabolite were: stimulation of angiogenesis, regeneration of infarct scars and breakthrough in cardiomyopathy rebuilding as well as sparing beneficial influence on post-infarcted rats’ heart myocardium achieved by the coronary flow increase without considerable heart rate elevation.

On the contrary, although chronic therapy with captopril improved the hemodynamic parameters (e.g. coronary flow increase, preload pressure decrease) in general, the histopathological study proved progressive post-ischemic cardiomyopathic process at the same time. We conclude, therefore, that when compared to captopril, M-2 presented itself as a more attractive agent in the long-term post-treatment after MI.

## Supporting information

S1 Table Individual values of measured biochemical parameters (row data). (XLSX)

S2 Table Hemodynamic parameters of post-infarcted rats hearts measured during 60 minutes of working heart experiment (row data). (XLSX)
